# Variation in uterus position prior to brachytherapy of the cervix: A case report

**Published:** 2017

**Authors:** MT Georgescu, R Anghel

**Affiliations:** * “Carol Davila” University of Medicine and Pharmacy, Bucharest, Romania; ** “Prof. Dr. Alexandru Trestioreanu” Institute of Oncology, Bucharest, Romania

**Keywords:** uterus, cancer brachytherapy, position, image-guided

## Abstract

**Rationale:** brachytherapy is administered in the treatment of patients with locally advanced cervical cancer following chemoradiotherapy. Lack of local anatomy evaluation prior to this procedure might lead to the selection of an inappropriate brachytherapy applicator, increasing the risk of side effects (e.g. uterus perforation, painful procedure ...).

**Objective:** To assess the movement of the uterus and cervix prior to brachytherapy in patients with gynecological cancer, in order to select the proper type of brachytherapy applicator. Also we wanted to promote the replacement of the plain X-ray brachytherapy with the image-guided procedure.

**Methods and results:** We presented the case of a 41-year-old female diagnosed with a biopsy that was proven cervical cancer stage IIIB. At diagnosis, the imaging studies identified an anteverted uterus. The patient underwent preoperative chemoradiotherapy. Prior to brachytherapy, the patient underwent a pelvic magnetic resonance imaging (MRI), which identified a displacement of the uterus in the retroverted position.

**Discussion:** A great variety of brachytherapy applicators is available nowadays. Major changes in uterus position and lack of evaluation prior to brachytherapy might lead to a higher rate of incidents during this procedure. Also, by using orthogonal simulation and bidimensional (2D) treatment planning, brachytherapy would undoubtedly fail to treat the remaining tumoral tissue. This is the reason why we proposed the implementation of a prior imaging of the uterus and computed tomography (CT)/ MRI-based simulation in the brachytherapy procedure.

**Abbreviations:** MRI = magnetic resonance imaging, CT = computed tomography, CTV = clinical target volume, DVH = dose-volume histogram, EBRT = external beam radiotherapy, GTV = gross tumor volume, Gy = Gray (unit), ICRU = International Commission of Radiation Units, IGRT = image guided radiotherapy, IM = internal margin, IMRT = image modulated radiotherapy, ITV = internal target volume, MRI = magnetic resonance imaging, OAR = organs at risk, PTV = planning target volume, QUANTEC = Quantitative Analyses of Normal Tissue Effects in the Clinic

## Introduction

Cervical cancer is one of the most common malignancies in developing countries [**1**]. For the locally advanced cervical cancer, concurrent chemoradiotherapy has been associated with an improvement in terms of overall and progression-free survival [**2**,**3**], becoming a standard for most therapeutic protocols in the preoperative or definitive setting [**4**,**5**]. Regarding the radiological treatment, external beam radiotherapy (EBRT) plays an important role in the treatment of cervical cancer, in time techniques like IMRT and IGRT [**6**] overcoming the classical “4-field box” technique. Although with these advanced techniques the radiation treatment became more conformal and therefore better tolerated, for the advanced stage patients, the best option in terms of radiotherapy is a combination of EBRT and brachytherapy [**7**,**8**]. CT/ MRI-based simulation have increased significantly worldwide, but most brachytherapy centers still rely on traditional plain X-ray imaging for treatment planning [**9**]. With this case report, we are strongly sustaining the use of high-technology simulation procedures for the brachytherapy of the uterine cervix.

## Methods and results

**Fig. 1:**
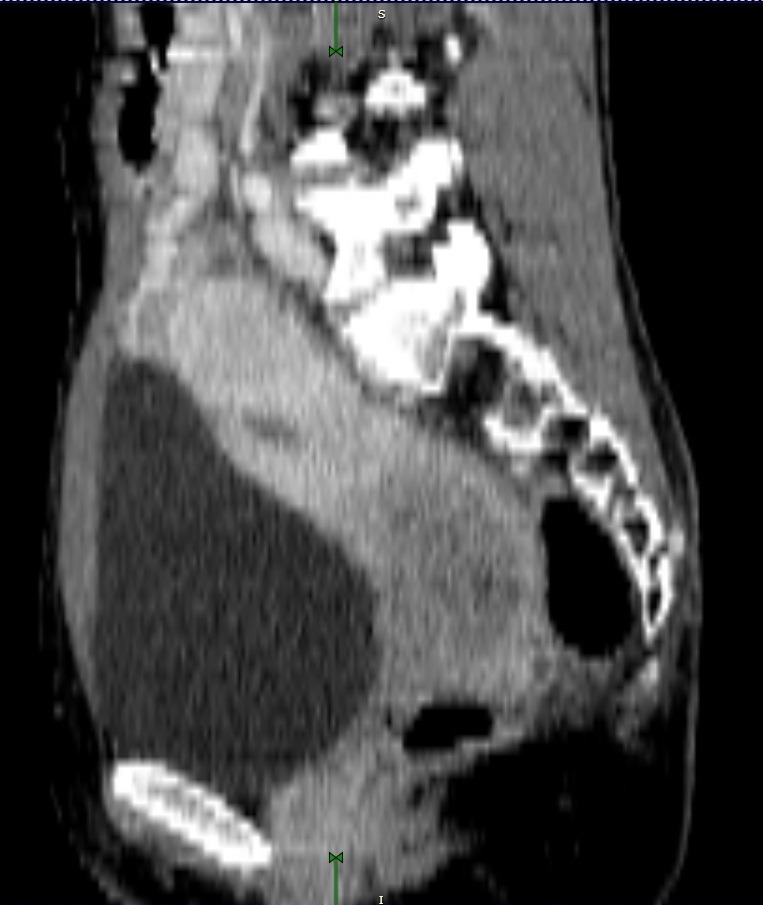
CT sagittal view of the patient’s pelvis before EBRT (anteverted position of the uterus)

 A 41-year-old patient was referred to the gynecology service after an evaluation of an abnormal vaginal bleeding, back pain, and pollakiuria. Pelvic examination identified a 3 cm polypoid cervical lesion with the invasion of the upper third of the left vaginal wall. Also, both parametria were found to be involved in their medial half. A tumoral biopsy was taken, thus confirming the presence of a cervical squamous carcinoma. The patient underwent a thorax-abdominal-pelvic CT scan that identified an anteverted uterus (**Fig. 1**), presenting a large tumoral mass, without a well-defined border with the rectum and the urinary bladder. No signs of tumoral invasion were found at cystoscopy and rectoscopy, only a bulging in the trigone area. Also, enlarged lymph nodes (max. 1 cm) were seen in the left obturator and right common iliac groups. No secondary tumors were found, therefore the cancer was staged IIIB [**8**]. 

The patient was referred to the oncology department and the multidisciplinary board decision was radiotherapy with weekly platinum radio sensitizing agent. CT simulation was performed, by using a 3 mm slice thickness. Intravenous and oral contrast was used. At simulation, the patient presented with full bladder and empty rectum, and she was instructed to maintain these values at each treatment session. Target volumes and organs at risk (OARs) were contoured on the CT images. The outer wall was contoured for the OARs. The bowel loops were contoured individually. Because at the CT scanning, the tumoral tissue was difficult to be differentiated by the cervical tissue, no gross tumor volume (GTV) was contoured. Clinical target volumes (CTV) were contoured. One of them was defined as CTV_45 and included the pelvic lymph nodes, uterus, parametria, annexes, and the upper 2/ 3 of the vagina. The second target volume was defined as CTV_54 and included the uterus, parametria, annexes and the upper 1/ 3 of the vagina.

**Fig. 2:**
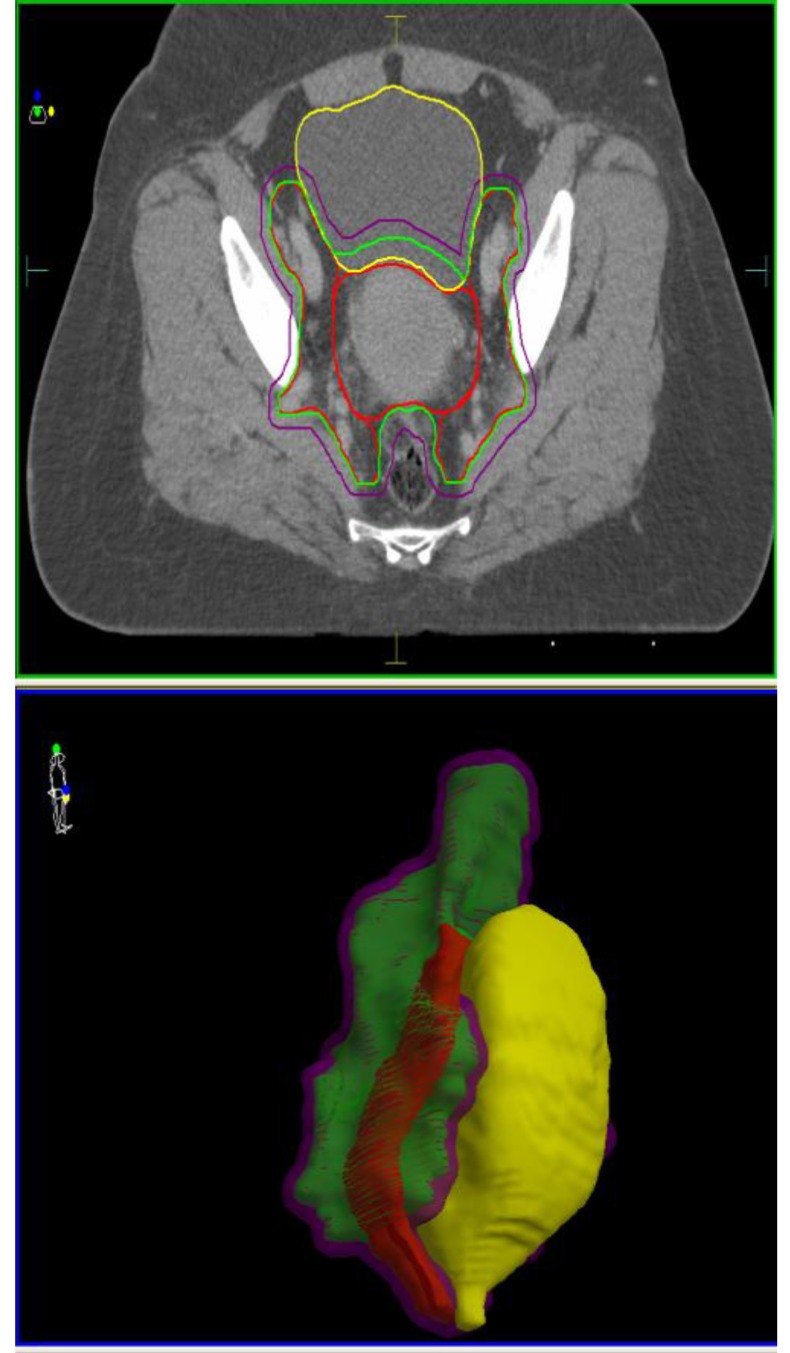
EBRT target volumes contoured according to ICRU62 recommendations (CTV-red, ITV-green, PTV-violet) and their relationship with the rectum (brown) and urinary bladder (yellow)

 Contouring was done in accordance with the ICRU62 Protocol [**10**] recommendations; therefore, the internal target volumes (ITV) were obtained for both CTV’s by adding a 1 cm internal margin (IM) into the urinary bladder. Planning target volumes (PTV), PTV_45 and PTV_54 resulted by adding a 0.5 cm margin around the ITV_45 and ITV_54 respectively (**Fig. 2**).

**Fig. 3:**
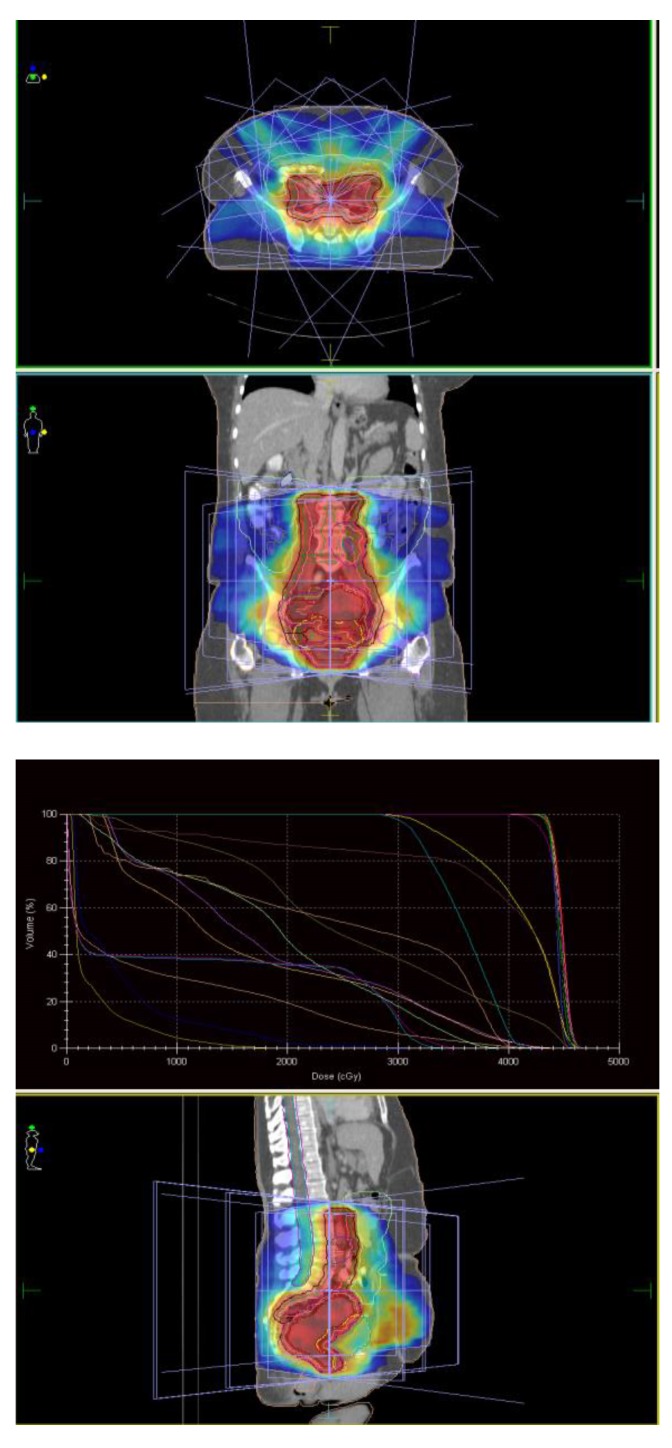
EBRT treatment planning by using the 7-field IMRT technique

Radiotherapy was administered with a preoperative intent; therefore, treatment was initially prescribed to a total dose of 4500 cGy/ 25 fractions/ 5 weeks to the PTV_45, followed by a boost of 900 cGy/ 5 fractions/ 1 week to the PTV_54. Radiotherapy planning was performed by using the inverse planning approach and xiO planning system, resulting in a 7-field treatment (**Fig. 3**). Plan-summing function was applied to evaluate the cumulative dosimetric data. 93% and 95% of the prescribed dose coverage on the dose-volume-histogram (DVH) was obtained for PTV_45 and PTV_54, respectively. The dose maximum was of 5686 Gy (105,3%) and was located within the PTV_54. The QUANTEC [**11**] dosimetric recommendations were achieved for the OARs. 

 Radiotherapy treatment was performed daily, with the patient in supine position, setup verification being made by a weekly megavoltage portal imaging. Cisplatin (40 mg/ m2) was administered for 5 cycles concomitantly weekly.

**Fig. 4:**
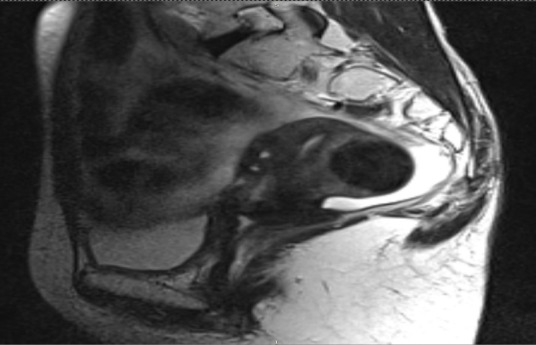
MRI sagittal view of the pelvis of the patient at the end of EBRT (retroverted position of the uterus)

 Treatment was completed with fair tolerance and was followed by pelvic MRI scan. This identified a significant reduction of more than 50% of the tumor mass, but also a change of the local anatomy, the uterus being retroverted (**Fig. 4**).

## Discussion

 Most brachytherapy studies [**12**-**15**] focus on the introduction of image-guided brachytherapy procedure for the treatment of cervix cancer, making a comparison between CT/ MRI-based volumetric calculations and the X-ray reference point estimates. Although there is currently a wide variety of brachytherapy applicators, literature data referring to the changes in the uterus position following EBRT and its dosimetric influence are limited. In 2005, Mayr et al. [**16**] conducted a study on the brachytherapy management by an ultrasound-guided applicator placement, which resulted in an acceptable outcome and complication rates. Considering that currently the tandem and ovoid applicator is still the most used worldwide [**17**,**18**], Small W. et al.’s literature review [**19**] recommend pelvic high performance imaging techniques before the brachytherapy applicator selection, in order to reduce the high rate of uterus perforation. Recently published data suggested [**20**] that the uterus position has an influence not only in terms of accidents, but also dosimetric, and in terms of toxicity, to the local normal tissues. 

## Conclusion

 CT/ MRI-based image-guided brachytherapy become necessary in order to have a better dosimetric assessment of the treatment plan and a valid prediction of their side effects.

Our clinical case highlighted the changes in the uterus position during radiotherapy. Therefore, in order to avoid unexpected treatment accidents (e.g. uterus perforation) we recommend the implementation of high performance imaging techniques in the simulation stage of the cervix cancer brachytherapy.

**Sources of founding**

This work received financial support through the project entitled “CERO – Career profile: Romanian Researcher”, grant number POSDRU/159/1.5/S/135760, co-financed by the European Social Fund for Sectoral Operational Programme Human Resources Development 2007-2013.

**Disclosures**

Authors declare that there is no conflict of interest regarding the publication of this paper.
